# Functional MRI activation of the nucleus tractus solitarius after taste stimuli at ultra-high field: a proof-of-concept single-subject study

**DOI:** 10.3389/fnut.2023.1173316

**Published:** 2023-10-26

**Authors:** Antonietta Canna, Elena Cantone, Anne Roefs, Sieske Franssen, Anna Prinster, Elia Formisano, Francesco Di Salle, Fabrizio Esposito

**Affiliations:** ^1^Department of Medicine, Surgery and Dentistry, "Scuola Medica Salernitana", University of Salerno, Baronissi, Italy; ^2^Faculty of Psychology and Neuroscience, Maastricht University, Maastricht, Netherlands; ^3^Center for Magnetic Resonance Research, University of Minnesota, Minneapolis, MN, United States; ^4^Section of ENT, Department of Neuroscience, Reproductive and Odontostomatological Sciences, "Federico II" University, Napoli, Italy; ^5^Biostructure and Bioimaging Institute, National Research Council, Napoli, Italy; ^6^Department of Diagnostic Imaging, University Hospital "San Giovanni di Dio e Ruggi D'Aragona", Salerno, Italy; ^7^Department of Advanced Medical and Surgical Sciences, University of Campania "Luigi Vanvitelli”, Napoli, Italy

**Keywords:** nucleus tractus solitarius, BOLD, fMRI, taste, chemosensory pathways

## Abstract

Using ultra-high field (7 Tesla) functional MRI (fMRI), we conducted the first *in-vivo* functional neuroimaging study of the normal human brainstem specifically designed to examine neural signals in the Nucleus Tractus Solitarius (NTS) in response to all basic taste stimuli. NTS represents the first relay station along the mammalian taste processing pathway which originates at the taste buds in the oral cavity and passes through the thalamus before reaching the primary taste cortex in the brain. In our proof-of-concept study, we acquired data from one adult volunteer using fMRI at 1.2 mm isotropic resolution and performed a univariate general linear model analysis. During fMRI acquisition, three shuffled injections of sweet, bitter, salty, sour, and umami solutions were administered following an event-related design. We observed a statistically significant blood oxygen level-dependent (BOLD) response in the anatomically predicted location of the NTS for all five basic tastes. The results of this study appear statistically robust, even though they were obtained from a single volunteer. The information derived from a similar experimental strategy may inspire novel research aimed at clarifying important details of central nervous system involvement in eating disorders, at designing and monitoring tailored therapeutic strategies.

## Introduction

Understanding the mechanisms underlying the cerebral processing of taste is fundamental for the study of eating disorders. Along the gustatory pathway, the functional role of the nucleus of the solitary tract (NTS) in taste processing is less studied in humans, mostly due to the difficulty in delineating this region *in-vivo* with MRI and, as a result, in analyzing the functional MRI (fMRI) signal induced by a gustatory stimulation. In this work, using 7 T ultra-high field (UHF) fMRI we aimed at detecting and localizing the gustatory activation response of the NTS evoked by the exposure to five basic tastes.

The role of the gustatory neural processes is not only to relay information on the quality, concentration, and (un)pleasantness of a substance but also to trigger the mechanisms of food intake regulation (1). The gustatory pathway originates from the oropharyngeal cavity and ultimately reaches the cerebral cortex. As the first relay station of the gustatory pathway in the brainstem (2), the gustatory nucleus is located in the rostral part of the NTS, within the medulla. In the mammalian central gustatory pathway, neural signals travel through axonal fibers of the facial, glossopharyngeal and vagus nerves (3–5), up to the thalamus, and, from there, directly to the insula and the operculum in the frontal lobe of the cerebral cortex (6, 7). In addition, a secondary cortical taste area is present in the caudolateral orbitofrontal cortex, where neurons respond to combinations of visual, somatic, olfactory, and gustatory sensory stimuli (8). The role of the NTS is therefore crucial to relay the gustatory and visceral information conveyed by chemo-responsive cranial nerves, to both promptly identify food as potentially poisonous or harmful to the body (9) and to regulate the feeling of satiety.

Unfortunately, NTS activity has proven to be hard to detect with fMRI, suggesting that UHF fMRI and special imaging procedures are required to detect gustatory neural responses at this level of the pathway, especially in pathologic conditions (10). The rostral part of the NTS is indeed theorized to play a role in integrating gustatory signals from taste receptors in the mammalian central gustatory pathway, enabling the discrimination among taste qualities (4) and intensities (3). Moreover, according to the temporal coding theory, the temporal dynamics of NTS activity are also crucial to detect differences among functional responses evoked by different tastes, considering that visceral and homeostatic signals also reach this place (5).

Although UHF fMRI has contributed greatly to the understanding of brain activity during gustatory perception, and of how these activities affect eating behavior (8, 11, 12), further investigations are still required. Most importantly, it remains crucial to dissociate the bottom-up sensory activation from the top-down perceptual response effects. A recent fMRI study on rats has shown that NTS is involved in the processing of both visceral and taste information, whereas the anterior insula is involved in the processing of taste oro-sensory signals (13).

More recently, the NTS structure and its connectivity have been studied in humans using 3 T and 4 T MRI scanners (14), the latter with the injection of a single taste (sucrose) solution. Particularly, the 3 T study of Forstenpointner et al. (15) assessed the connections to and from the NTS in a group of 20 healthy subjects using both structural and functional connectivity analyses, emphasizing the important role played by NTS in the integration of viscerosensory information.

Further evidence of the important role played by the NTS in taste processing has been provided by Hoogeveen et al. (16). In this study, the authors measured fMRI BOLD activation across several brain areas of the central gustatory pathway, including NTS, but did not report any significant effects that could be related to the quality or concentration of the injected tastants. On the other hand, to the best of our knowledge, no studies at 7 T have been performed to specifically detect the BOLD activation in the NTS due to gustatory stimulation.

One of the reasons explaining the lack of taste-related NTS fMRI BOLD activity in some previous reports could be that the acquisition and analysis of such a small and deep brain region might require an isotropic voxel of ~1 mm (or less) and the application of spatial smoothing kernels of ~2 mm (or less) (17). These are very strict requirements for most clinical high-field (18) MRI scanners. As a demonstration, a recent study at 7 T has reported a significant BOLD response in the NTS after a cold pressor test (19), showing the feasibility of recording BOLD activity from this region at UHF. Moreover, whereas 9.4 T fMRI (17) has revealed both insular and NTS activity in animals after repeated tasting of sucrose solutions (13), similar evidence is still lacking in humans.

However, due to the complex nature of gustatory experiments, it is not yet clear how to design a pilot study in terms of paradigm, amount of delivered tastant, sequence settings, and data processing, and, therefore, a proof-of-concept study remains mandatory, considering also the costs of 7 T experiments. Accordingly, we conducted a proof-of-concept study in a single subject on a UHF scanner with the specific aim to test the feasibility of measuring NTS BOLD fMRI response to taste stimuli at 7 T.

## Materials and methods

### Participant

Data of one adult volunteer participant (26 years old female, BMI 20.8 kg/m^2^, right-handed, nonsmoker, not reporting any olfactory, gustatory, neurological, or psychiatric disorder, and free from the use of any medication) were acquired for this study. The Ethical Committee of the Faculty of Psychology and Neuroscience at Maastricht University granted approval for this study (protocol number ERCPN 159_15_12_2015_S6) and the participant signed a written consent.

### fMRI parameters

**Figure 1:**
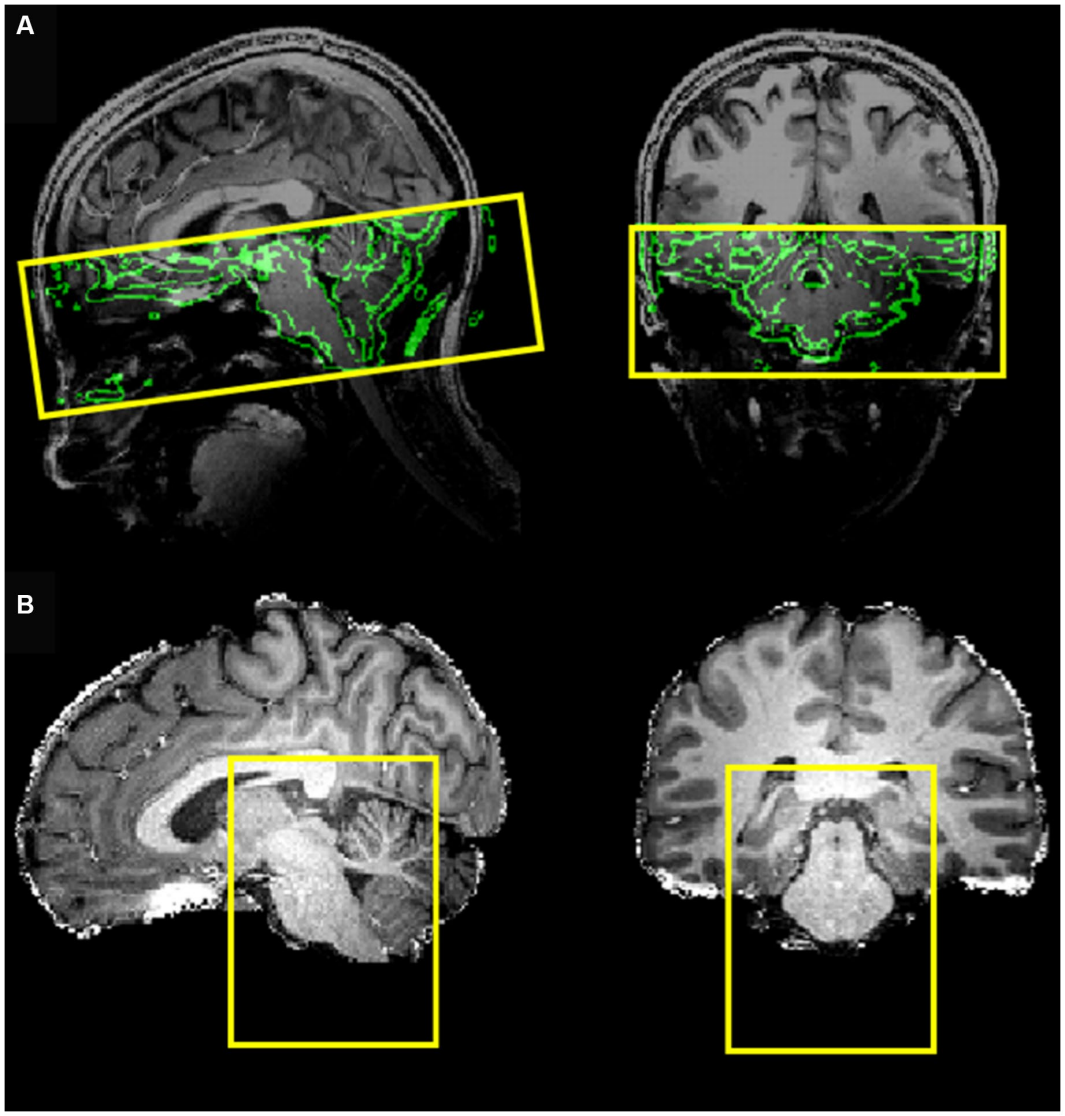
**(A)** Field of view of the EPI acquisition (coverage) with the border of the brain (obtained from the T1-weghted image) shown in the background. **(B)** Bounding box covering the entire brainstem, selected to reduce the processing region of interest.

MRI data acquisition was performed on a Siemens MAGNETOM 7 Tesla MRI scanner with a 32-channel RF coil. The acquisition protocol included a three-dimensional T1-weighted Magnetization Prepared Rapid Gradient-Echo (MPRAGE) sequence (TR 3,100 ms, TE 2.52 ms, TI 1,500 ms, flip angle 5°, slice thickness 0.6 mm, matrix size 384 × 384, number of slices 192, voxel size 0.6 × 0.6 × 0.6 mm^3^), a three-dimensional proton density (PD) sequence (TR 1,440 ms, TE 2.52 ms, flip angle 5°, slice thickness 0.6 mm, matrix size 384 × 384, number of slices 192 and voxel size 0.6 × 0.6 × 0.6 mm^3^) and a BOLD fMRI sequence (gradient-echo EPI with multi-band (18, 20, 21) factor of 2, TE 19 ms, TR 1,500 ms, voxel size 1.2 × 1.2 × 1.2 mm^3^, 50 slices, 460 dynamic scans, matrix size 160 × 160, Field of View (FoV) 192 ×192 mm, direction of phase encoding acquisition AP, total scan duration 11 min and 30 s) followed by two identical series with opposite phase encoding (AP, PA) and 5 dynamic scans (for EPI distortion correction). The FoV of this acquisition was precisely determined to cover the brainstem up to the NTS (Figure 1A). To optimize the computational burden of the functional data, processing was restricted to a reduced bounding box covering the entire brainstem (Figure 1B).

### fMRI stimulation protocol

During fMRI acquisition, three shuffled injections of sweet (sucrose), bitter (quinine hydrochloride), salty (sodium chloride), sour (citric acid) and umami (glutamate) solutions were administered to the supine subject, in the middle of the mouth, via a gustometer consisting of independently programmable BS-8000 syringe pumps (Braintree Scientific, Braintree, MA) automatically controlled by a stimulation computer. During the experiment, the gustometer was placed in the console room and, thus, the tastants were administered to the subject at room temperature. We instructed each subject to swish and immediately swallow each taste upon its arrival in the mouth. The experimental paradigm consisted of an event-related design based on the injections of all basic tastants and individually chosen artificial saliva (used for taste nonspecific rewashing purposes) (12). During the experiment, each tastant and rewashing injections were anticipated by a visual cue, that is a picture displaying “Taste” or “Rewash” in white characters on a black background which was maintained during the intervals between two consecutive injections. A taste recognition phase was inserted between the two events, consisting of a picture displaying the correspondence between buttons and tastes. At each presentation, the participant was asked to recognize the incoming taste by pressing the corresponding button.

**Figure 2:**
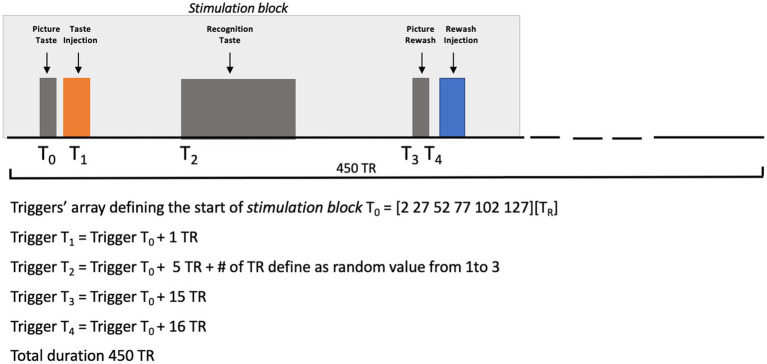
Picture Taste: the word “TASTE” was presented on a video display unit (connected to a back-projection screen in the MRI room) for 1,000 ms. Taste Injection: the pump of the gustometer was activated for 1,400 ms and 0.7 mL of solution of a specific taste was delivered. Recognition Taste (with a jittered delay between 6 and 8 TR after the prior taste injection): a legend showing the correspondence between buttons (coding between 1 and 6) and the 5 tastes plus saliva was presented on a video display unit (connected to a back-projection screen in the MRI room) for 6,500 ms. Picture Rewash: the word “REWASH” was presented on a video display unit (connected to a back-projection screen in the MRI room) for 1,000 ms. Rewash Injection: two pumps of the gustometer were activated for 1,400 ms and a total volume of 1.4 milliliter of saliva was delivered. The single block lasted 11.5 min.

All basic tastants (sweet = sucrose, bitter = quinine hydrochloride, salt = sodium chloride, sour = citric acid, and umami = glutamate) were prepared in distilled water, whereas the artificial saliva was prepared as described in O’Doherty et al. (7). All taste solutions were injected in volumes of 0.7 mL delivered over 1.4 s, while the artificial saliva was injected with two separate pumps, each delivering volumes of 0.7 mL over 1.4 s. Taste concentrations, that were preliminarily selected by the subject in a laboratory screening, were 117, 0.125, 100, 20, and 100 mM, respectively, for sweet, bitter, salty, sour, and umami. Before starting the scanning, the subject was asked about his possible (dis)comfort in the magnet and, particularly, whether she was feeling nausea or a metallic taste. The synchronization of stimulus delivery with fMRI scanning was controlled via a MATLAB custom script (The MATHWORKS Inc., Natick, MA, USA) including functions from Psychotoolbox (22–24). Figure 2 reports a graphical representation of the experimental design.

### fMRI preprocessing

To detect the functional activity of the NTS, MRI image data preprocessing and statistical analyses were performed across the entire brainstem using BrainVoyager (Brain Innovation, Maastricht, The Netherlands, www.brainvoyager.com), SPM12,^1^ and fMRI Software Library (FSL; http://fsl.fmrib.ox.ac.uk/fsl). The anatomical T1w images were preliminarily divided by the co-registered PD images to reduce intensity bias. To further improve the quality of the obtained anatomical reference, an additional bias correction was performed using SPM, prior to skull stripping before registration of the fMRI images. The fMRI time series were corrected for head movement-related artifacts and for the differences in slice scan times using standard procedures in BrainVoyager and for geometrical distortion via the TOPUP tool of FSL (25, 26). Subsequently, the fMRI data were high-pass filtered in time (cut-off 128 s) to reduce linear and nonlinear trends in the time courses and slightly smoothed in space with an isotropic Gaussian kernel of 2 mm full-width half maximum.

### fMRI data analyses

Preprocessed EPI time series were analyzed using the general linear model (GLM), by entering stimulus timings and choosing double-gamma functions, to define both event-related and box-car predictors. The latencies of taste injections were used to define the event-related predictors of interest. The latencies of visual cues (anticipating the injections), rewashing phases, and recognition-related button responses, as well as the six motion parameters, were included as confound predictors. A correction for serial correlation was applied using a fit-refit procedure for the GLM with a second order auto-regressive model applied to the GLM residuals. Z-transformation was applied to all predictors and voxel time courses before the GLM fitting.

From the GLM, a *t*-statistic contrast map was generated for the main effects of all taste stimuli (vs. artificial saliva). A statistical threshold of *p* < 0.05 (Bonferroni corrected across all voxels of the selected bounding box) was applied to determine possible clusters of significant activation. Eventually, these were converted to regions of interest, and an event-related averaging of the BOLD response time courses was performed with respect to triggers of taste-related stimuli and (for comparison between sensory modalities) visual cues. The event-related response was computed by extracting the mean regional BOLD signal in time intervals from 2 s before (−2 s) to 16 s after (+16 s) each stimulus repetition, with % signal change normalization to baseline (−2, 0 s). To enable comparisons with the results of a previous meta-analysis [see (10)] reporting peak MNI coordinates for the NTS, we also transformed the images to the MNI space.

## Results

**Figure 3:**
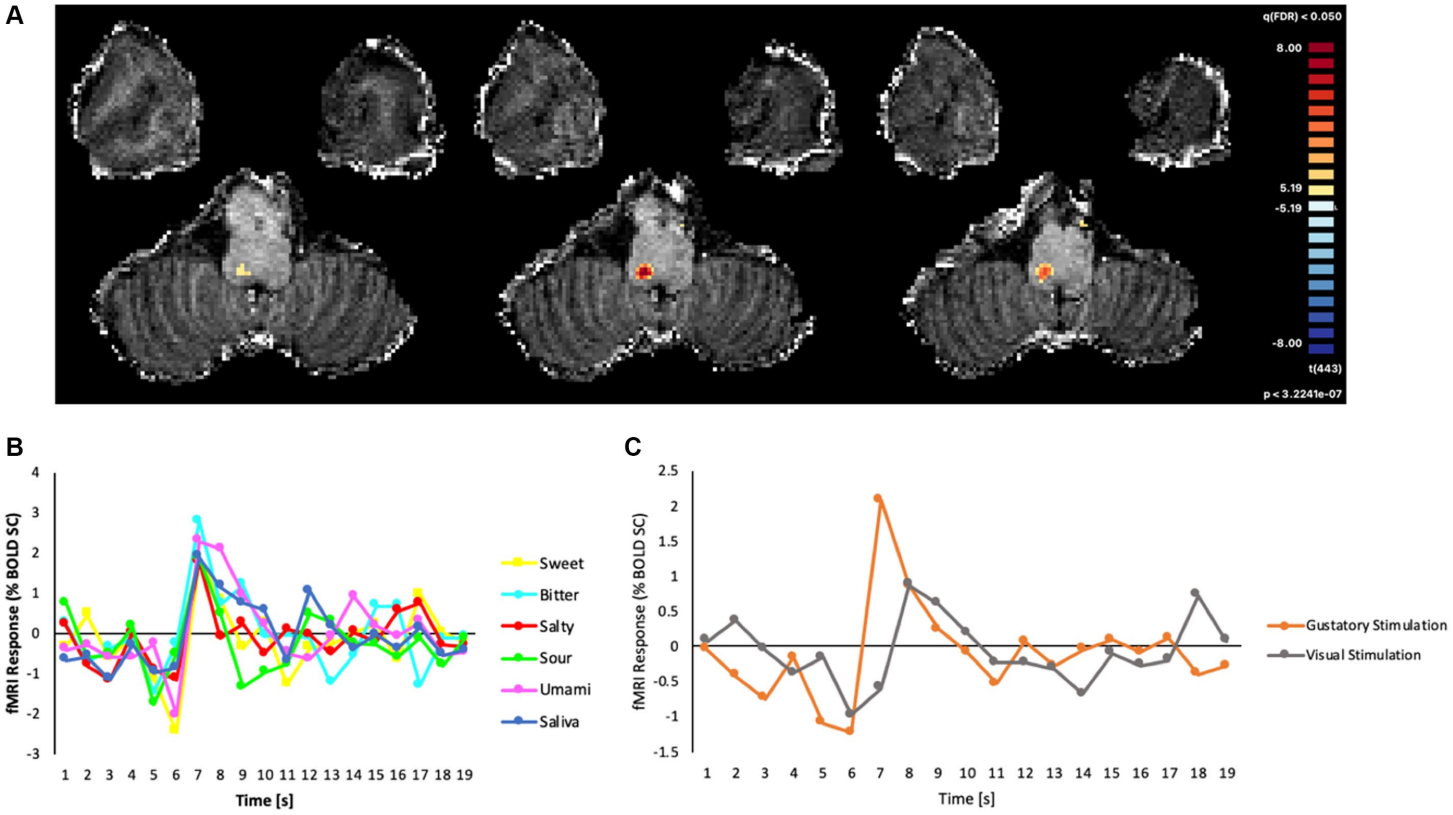
**(A)** Map of significant activation from the GLM analysis in the brainstem, overlaid on the T1w anatomical image, **(B)** Average fMRI response (% BOLD signal change) in the cluster of significant main effects evoked by all gustatory stimuli. **(C)** BOLD percent signal change in the same cluster to all gustatory stimuli (mean) and to non-gustatory (visual) stimuli.

As the field of view was restricted to the brainstem and tailored to the anatomy of the subject, we were able to verify that the ultra-high-resolution (1.2 mm isotropic) EPI acquisitions effectively covered the NTS. From the voxel-wise GLM analysis, we found that the event-related BOLD responses (estimated as the percent signal-change of the signal triggered by the gustatory or visual stimulation), produced a robust cluster of functional activation (peak: *t* = 8.22, cluster size: 40 voxels, *p* < 0.05; Bonferroni corrected) for all basic tastes (sweet, bitter, salty, sour, umami), and this cluster was located within the dorsal portion of the medulla oblongata (Figure 3A). In the MNI space, the area of the cluster was maximal at slices z = −54, −55, −56.

The *post hoc* event-related temporal analysis of the averaged BOLD fMRI response in this cluster revealed maximal fMRI responses to all gustatory stimuli between 4 and 7 s after the stimulus presentation, the highest percent signal change of all conditions (including saliva) being observed for bitter (2.78%) and the lowest for salty (1.79%) tastes (Figure 3B). In contrast, the event-related fMRI responses detected for non-gustatory (e.g., visual) stimuli were significantly lower than the ones produced by the gustatory stimulations (Figure 3C).

## Discussion

We presented a proof-of-concept study on a single human subject aimed at verifying the technical feasibility of an *in-vivo* non-invasive measurement of BOLD fMRI signals in the NTS, in response to gustatory stimuli, using a 7 T MRI scanner. By performing a tailored acquisition of the anatomical area of interest, and using experimental settings optimized for event-related gustatory stimulation, we were able to report the first functional gustatory activation of the human brainstem using UHF (7 T) fMRI, in response to all basic tastes (sweet, bitter, salty, sour, and umami). In the MNI space, the location of the cluster nicely matched the NTS anatomical location illustrated in a previous structural 7 T MRI study (10) where the anatomical dissection of the NTS was also achieved through specialized procedures starting from a preliminary meta-analysis [see (Figure 3) in Priovoulos et al. (10) for comparison]. In our study, the use of UHF fMRI has been crucial to detect the precise location of the NTS response, enabling the measurement of a significant event-related BOLD response to gustatory stimuli with both high spatial resolution and reasonably short acquisition times. The analysis of the measured fMRI time series was performed in the native voxel space and only included minimal spatial smoothing, respectively to preserve the original spatial resolution and to limit the impact of physiological noise sources from surface vessels (27). We chose a voxel size that approximately matched the size of NTS on the axial plane (28) but was still able to keep to a reasonable level the impact of physiological and thermal noise, the latter being known to increase with magnetic field and often becoming a major limiting factor at lower resolutions [e.g., 3 mm (29)]. Indeed, in this proof-of-concept study, BOLD signal changes appear strongly related to taste stimuli, including control artificial saliva, rather than to other sources.

The complex nature of gustatory fMRI experiments generally imposes several choices in terms of both acquisition and experimental settings and alternative options would be available. Besides different criteria for defining the slice orientation and choosing the sequence parameters, a block design (with longer periods of injection) might in principle elicit stronger signal changes compared to an event-related design (with short periods of injection) but this would also make it far more difficult to precisely control the amount of taste to deliver and would likely introduce saturation effects. Nonetheless, the chosen setup appeared feasible and effective to elicit a significant activation in the NTS, which was detectable at a single-subject level.

Neural activation elicited by gustatory stimuli has been observed in humans across cortical and subcortical regions, including the insula, cingulate cortex, thalamus, and amygdala in many fMRI experiments (8, 11, 12) but, so far, no published studies have reported a functional response to gustatory stimuli in the NTS at UHF-7 T. This is presumably because tailored functional imaging acquisitions with UHF MRI, albeit more difficult to standardize, are probably strictly needed for studying the NTS, as explicitly noted recently (10). As a result, while NTS responses to gustatory stimuli have been observed in animals (4), very little is known in humans, also due to the difficulty in performing non-invasive functional studies of this nucleus, given its small dimension and location.

The NTS receives input from the oropharyngeal cavity through branches of the facial, glossopharyngeal, trigeminal, and vagus nerves. In particular, the anterior oral cavity terminates in the rostral portion of the NTS, whereas the posterior oral cavity terminates more caudally. In the NTS, the pharyngeal and laryngeal receptive fields terminate even more caudally. Thus, how the NTS processes taste-specific information in humans, and what would be the role of NTS in temporally integrating taste-relevant information, remains far from being understood and requires novel *ad hoc* experimental strategies (15). In particular, it remains to be understood if different tastes are processed differently in the NTS, in which case an even smaller scale would be needed to identify taste responsive neuron subsets (30).

Our experimental setting encourages moving to UHF since, according to our results, gustatory signals from the periphery, including saliva, elicit a significant neural response in the NTS. Even though saliva is often used as a neutral gustatory stimulus, it is possible that this stimulus also triggers the activation of the thalamocortical and higher-order cortical networks which is ultimately responsible for taste-related perception and behaviors (1). In contrast, a different type of stimulus, such as the visual cue used in the stimulation paradigm, did not induce a similar BOLD response in the same NTS area, thus ruling out similar effects from other physiological or sensory sources. Indeed, compared to the gustatory activation, the event-related response to visual stimulation appeared both delayed in time and reduced in amplitude (peak ~1% signal change Figure 3C).

Another possible explanation for the NTS activation to the tasteless solution could be that the mechanism of ingestion, while physically independent of the quality of incoming stimuli, could still contribute to a taste-related response in this structure, especially if the actual discrimination and/or the identification of the taste itself would occur in other stations of the central gustatory pathway, where the amplitude of the functional response would be specifically modulated by the taste quality. This hypothesis deserves further investigation.

UHF fMRI in animals (13) has employed oral taste stimulation and gastric distension to demonstrate NTS activation (31). Emerging evidence suggests that the brain receives rapid sensory cues from the gut independently of the oral stimulation and that NTS neurons would play a critical role in both maintaining homeostasis and in the processing of (sweet) stimuli (32–34). Thereby, the NTS activation to gustatory stimuli has important implications for eating disorders. Indeed, fMRI studies have shown that patients with anorexia and bulimia nervosa process adverse and pleasant stimuli in opposite ways, compared to healthy subjects (35). Nonetheless, whether these differences are mediated by differential NTS activation levels has never been probed so far.

Our proof of concept study has several limitations. First, it has been conducted on a single subject and, therefore, it needs to be confirmed in a pilot study on a number of subjects sufficient to ensure adequate statistical power for a group-level analysis (36). Second, our efforts were mainly focused on reaching an unprecedently high spatial resolution of the functional measurements, which we believe is mandatory to address the BOLD responses in such a small structure. However, we might have been only partially able to balance the higher signal and contrast-to-noise ratios at 7 T [as compared to 1.5 T or 3 T (27)] against the higher physiological and thermal noise and the presence of other sources of imaging artifacts, which are to be faced in UHF experiments. In addition, our single-subject analysis was conducted in the native voxel space of the fMRI acquisition with minimal spatial smoothing. Therefore, additional issues concerning anatomical normalization and spatial smoothing are to be investigated before pooling data from groups of subjects, as required for multisubject fMRI studies.

In our single-subject analysis, the activation that remained significant after the Bonferroni correction covered only one side of the brainstem. This unilaterality can be due to multiple factors. First, this could be due to the low power of our analysis (single-subject analysis and only 18 repetitions of taste stimuli), thereby only the dominant side of the functional activation in one hemisphere is detected. On the other hand, a unilateral pattern of gustatory stimulation is often seen also in the primary cortex where subjects typically exhibit variable activations in the left and/or the right insular cortex (6, 14). However, in order to better investigate the unilateral vs. bilateral activation of the NTS to gustatory stimuli, a complete study enrolling more subjects will be needed.

The last crucial aspect that needs to be addressed relates to the fact that the NTS might be sensitive to oral somatosensory stimulation in general, as previously observed in rats (37). In this case, some movement-related activity in this structure would be mixed with the neural effects resulting from gustatory stimulation. This would explain why the same amplitude of the BOLD signal change was observed here in response to the artificial saliva and the other basic tastes. However, it has been previously shown (38) that tasteless solutions can also induce a cortical (insular) response, similar to that produced by other tastants, and this response might be related to the expectation of taste. While this could have been the case in our experiment, our single-subject study would not allow addressing this issue definitely, albeit it represents an important starting point for future studies in larger populations.

In conclusion, despite all the above limitations, we believed that our proof of concept study might be a useful guide for the design of gustatory fMRI experiments and the acquisition of high-resolution fMRI images of the brainstem in response to gustatory stimulations.

## Data availability statement

The raw data supporting the conclusions of this article will be made available by the authors, without undue reservation.

## Ethics statement

The studies involving human participants were reviewed and approved by the Ethical Committee of the Faculty of Psychology and Neuroscience at Maastricht University (protocol number ERCPN 159_15_12_2015_S6). The patients/participants provided their written informed consent to participate in this study.

## Author contributions

AC, FS, EC, and FE: conceptualization. AC, AR, SF, and AP: methodology. AC, EF, and FE: formal analysis. AC, AR, SF, and EF: investigation. AR, EF, and FE: resources. AC, AP, and FE: writing—original draft preparation. AC, AP, AR, SF, EC, FS, and FE: writing—review and editing. All authors contributed to the article and approved the submitted version.

## Footnotes

^1^www.fil.ion.ucl.ac.uk/spm/software/spm12/
